# Imaging urolithiasis: complications and interventions in children

**DOI:** 10.1007/s00247-022-05558-6

**Published:** 2022-12-28

**Authors:** Magdalena Maria Woźniak, Joanna Mitek-Palusińska

**Affiliations:** Department of Pediatric Radiology, Medical University of Lublin, Al. Racławickie 1, 20-059 Lublin, Poland

**Keywords:** Calculi, Children, Computed tomography, Kidneys, Nephrolithiasis, Ultrasound, Urolithiasis

## Abstract

Urolithiasis affects people in all age groups, but over the last decades there has been an increasing incidence in children. Typical symptoms include abdominal or flank pain with haematuria; in acute cases dysuria, fever or vomiting also occur. Ultrasound is considered the modality of choice in paediatric urolithiasis because it can be used to identify most clinically relevant stones. Complementary imaging modalities such as conventional radiographs or non-contrast computed tomography should be limited to specific clinical situations. Management of kidney stones includes dietary, pharmacological and urological interventions, depending on stone size, location or type, and the child’s condition. With a very high incidence of underlying metabolic abnormalities and significant recurrence rates in paediatric urolithiasis, thorough metabolic evaluation and follow-up examination studies are of utmost importance.

## Introduction

Urolithiasis, which refers to accumulation of stones along the urinary tract, is a common condition affecting people in all age groups. Over the last decade or two, the incidence of urinary stone disease in children has increased significantly, with the highest rates in adolescent girls [1, 2]. The mean annual rate reported by Ward et al. [2] was 59.5 cases per 100,000 U.S. children, still infrequent compared to 217/100,000 in adult women and 299/100,000 in adult men [3].

The most common symptoms of urolithiasis include abdominal or flank pain and macro- or microhaematuria; nausea, vomiting or fever might coexist. Because of indeterminate symptoms, urolithiasis might be initially misdiagnosed, especially in newborns and infants, who often present to the emergency department with only irritability. Many children with kidney stones remain asymptomatic and are diagnosed incidentally on imaging examinations.

Underlying metabolic abnormalities are one of the most important risk factors of paediatric urolithiasis, identifiable in more than 50% of affected children. The most common metabolic aberrations are hypercalciuria (52–64%), hyperoxaluria, hypocitraturia and cystinuria [1, 4, 5]. Other risk factors include urinary tract infections (UTIs), urinary tract malformations and diversions, low fluid intake and high sodium intake.

As with the adult population, most paediatric kidney stones are composed of calcium oxalate and calcium phosphate. Other stones, including struvite or cystine, are less common. Urinary tract infections, as well as urinary tract malformations predisposing to UTI, including horseshoe kidney and duplex collecting system, can increase the risk of developing struvite stones [1, 5, 6].

Recurrence rates in paediatric patients are high, especially among children with metabolic abnormalities; recent studies showed 15% to 50% recurrence rates during the 3 years after treatment, indicating a necessity for follow-up examinations [1, 4, 7, 8]. Given the high incidence of metabolic aberrations combined with high recurrence rates in children, metabolic studies (including screening for acidosis, serum electrolytes, blood urea nitrogen, creatinine, serum phosphorous, magnesium, calcium and uric acid levels) should be performed in every case of kidney stones confirmed on diagnostic imaging [6].

The aim of this review is to provide an overview of imaging modalities and interventional treatment methods used in paediatric urolithiasis and to highlight specificity of diagnostics and treatment in these children.

## Diagnostic imaging

### Ultrasound (US)

Many imaging modalities can be useful in diagnosing urolithiasis, including US; conventional radiographs of the kidney, ureter and bladder (KUB); non-contrast CT; and magnetic resonance (MR) urography [9]. Unlike in the adult population, where non-contrast CT is considered the gold standard in the diagnosis of urolithiasis, US, being a non-ionizing, easily accessible and effective procedure, is recommended as the initial diagnostic method in children [1, 6, 10]. Although sensitivity and specificity of US depend on the physician as well as the machine and patient position, they have been reported to be as high as 67–90% and 95–100%, respectively [1, 11].

**Fig. 1:**
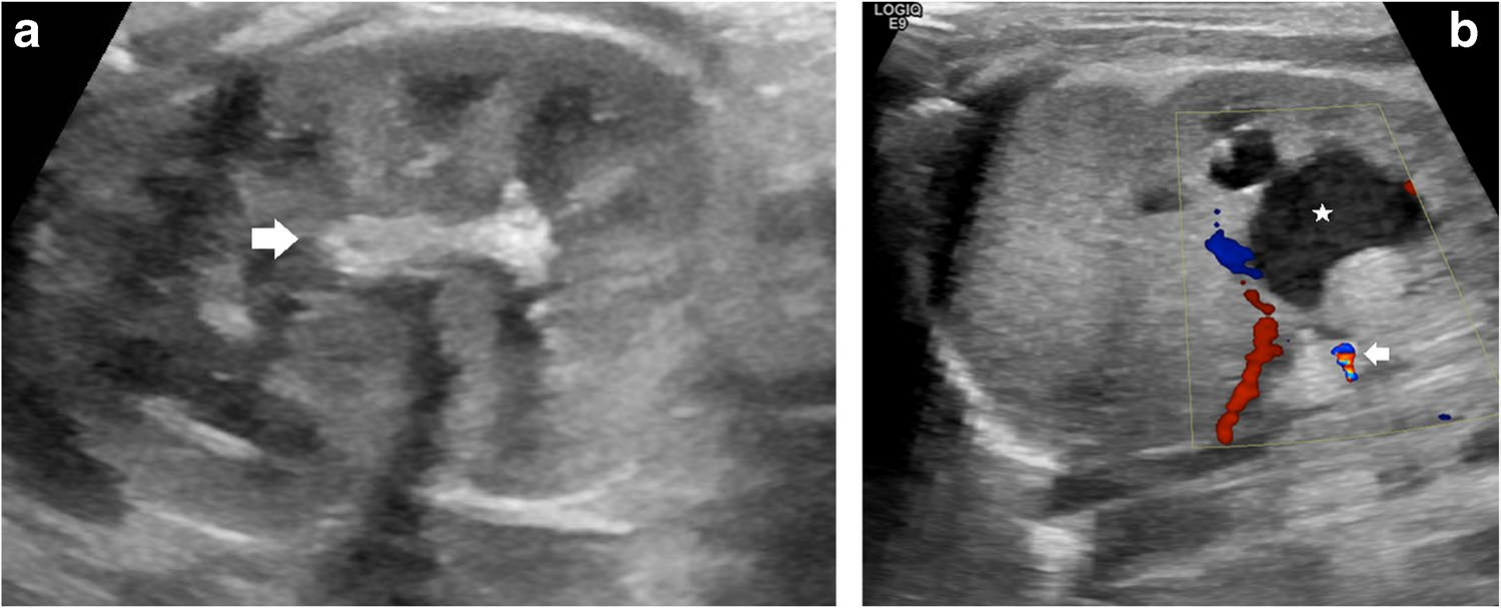
Drug-induced urolithiasis (post-diuretic) in a premature 35-week-old boy (born at 26 weeks of gestational age). **a** Sagittal US of the left kidney shows a large, calcified structure in the renal hilum (*arrow*), corresponding to a stone. **b** Follow-up US colour Doppler examination after 5 weeks. Transverse view of the kidney shows the stone to have dissolved, with formation of a calculus in the proximal part of the ureter visualised by virtue of the twinkling artifact (*arrow*). Obstruction of the ureter resulted in pelvicalyceal dilatation with echogenic concrement in the renal pelvis (*asterisk*). Case courtesy of Prof. Philippe Petit, Marseille, France

Ultrasound is used to visualise presence, size and location of stones, along with potential complications of urolithiasis. Typical US findings of urolithiasis include a hyperechogenic structure in the renal collecting system with or without posterior shadowing, depending on the size of the calculus. Pelvicalyceal dilatation, an indirect sign of obstruction, might also be seen (Fig. 1) [9, 12]. In chronic cases of severe obstruction, thinning of the renal cortex can occur.

**Fig. 2:**
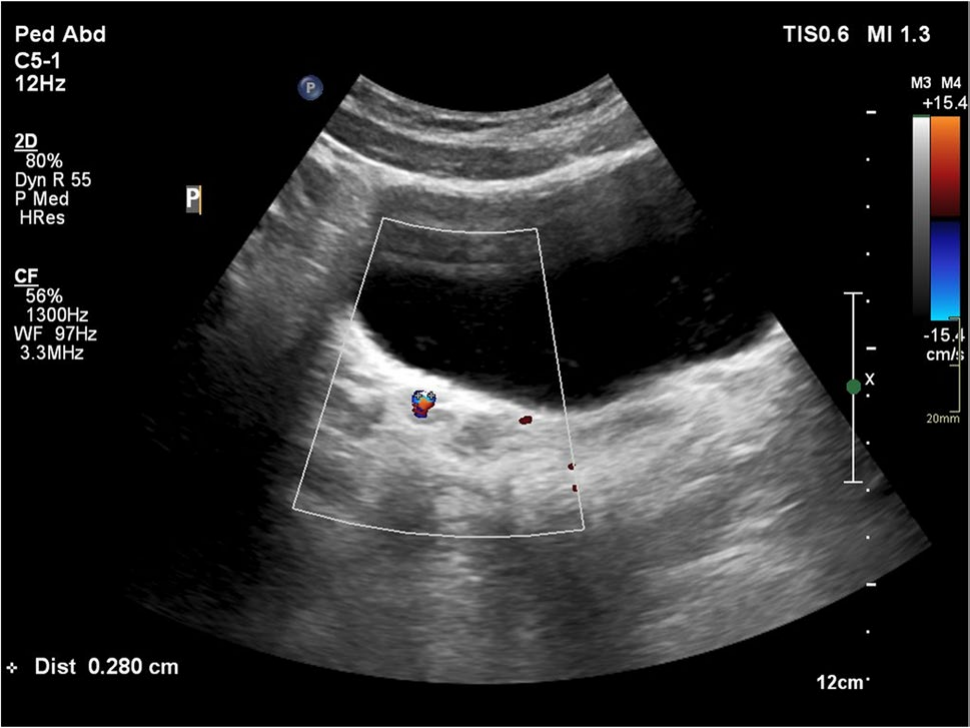
Colour Doppler axial image of the bladder of a 17-year-old girl who presented with right lower quadrant pain and haematuria shows a stone in the distal part of the right ureter. Twinkling artifact facilitates differentiation of the stone from other echogenic structures

Ultrasound should cover the urinary tract from the top of the kidneys to the bottom of the bladder. It ought to be performed in a well-hydrated, calm child, in both supine and prone positions, including curved and linear transducers. When cystolithiasis is suspected, the lateral decubitus position may be useful to confirm the nature and possible movement of an echoic structure in the bladder. When measuring identified calculi, it is suggested that the width of the acoustic shadow behind the stone, rather than the hyperechogenic line is measured, given the possible overestimation of the stone’s true size [12]. US efficacy can be improved by using colour Doppler, which may produce the “twinkling artifact,” defined as the appearance of alternating colours behind a reflective object. Twinkling artifact, reported to be seen in more than 80% of urinary stones, can help to confirm or exclude presence of a stone, especially in cases of uncertainty caused by the proximity of the echogenic renal sinus or its possible location near the ureterovesical junction (Fig. 2). To obtain best results, increase the pulse repetition frequency to suppress background colour signal and aim the beam at various angles to improve identification and visualisation of the artifact [13]. The focal zone should be located below the stone; moving it at or above the stone may weaken the artifact [14].

Other possible US findings in children with urolithiasis include weakening of the ureteric jet and elevation in renal resistive index resulting from obstruction, which might precede pelvicalyceal dilatation [15, 16].

A few possible mimics of kidney stones might arise on US, one of the most important being medullary nephrocalcinosis, referring to accumulation of calcium deposits in medullary pyramids. This condition may be differentiated from nephrolithiasis by the specific localisation of the echogenic foci outside the collecting system; early changes involving only the apices of the medullary pyramids do not result in acoustic shadowing. Other causes of hyperechogenic medullary pyramids or their apices include papillary necrosis, medullary sponge kidneys or renal infection. In neonates, Tamm-Horsfall protein accumulation resulting in transient pyramidal echogenicity must be considered. An echogenic, highly vascular benign tumour — angiomyolipoma — is another possible sonographic mimic of a kidney stone. It can be differentiated by its intraparenchymal or exophytic localization; acoustic shadowing is occasionally seen. Echogenic renal foci can also represent calcifications in renal vessels or in the cortex. It is important not to confuse a ureteric stent left in place following urological procedures for pathological echogenic foci. In cases of unclear US appearance of echogenic renal foci, the use of other imaging modalities such as non-contrast CT should be considered [17].

### Kidney, ureter, bladder (KUB) radiography

Kidney, ureter and bladder (KUB) radiography applied alone has low estimated sensitivity and specificity (57–69% and 76–82%, respectively) [10, 12]. Calculi may be obscured by bowel content due to inadequate bowel preparation, obesity or extrarenal calcifications. Moreover, not all stones are radiopaque. Visibility of a stone on a KUB radiograph depends on its composition — calcium-containing calculi are radiopaque; struvite or cystine are sometimes opaque; uric acid, medication or matrix stones are radiolucent and impossible to see on radiography [10, 12]. Exact location or signs of pelvicalyceal dilatation are undetectable on KUB radiographs; thus, a KUB radiograph is recommended only as a method additional to US [9, 12, 18]. Radiographic identification of stones missed on US is rare, and US alone is sufficient in most cases. Therefore, KUB radiography in combination with US should be reserved for specific clinical situations because the risk of ionising radiation exposure should be always balanced by possible benefits, especially in paediatric patients [1]. As reported by Marzuillo et al. [19], many children with symptoms and metabolic risk factors of urolithiasis are negative on US, KUB radiography and even CT; thus, the authors suggested repeat US examination 1–2 years later in children suspected of having urolithiasis with negative US and KUB radiography evaluations to detect possible calculi.

### Non-contrast computed tomography

Non-contrast CT is considered the gold standard for the diagnosis of urolithiasis in adults [18, 20]. It has the potential to visualise almost all types of calculi, even those located in the ureters; to define the exact localisation, size and shape of stones; and to reveal signs of pelvicalyceal dilatation or possible differential diagnoses [9]. Given all these advantages and its very high sensitivity (97–100%) and specificity (96–100%) in diagnosing urolithiasis, non-contrast CT would be considered the perfect method if not for its ionising radiation burden [10, 12]. The potential risk of developing radiation-associated malignancies contributed to creating similarly effective ultra-low-dose non-contrast CT protocols with reported mean effective radiation dose 0.8–2.5 mSv (with that of KUB radiography about 0.01–0.11 mSv), which have been implemented for detecting urolithiasis in both children and adults [9, 10, 21–23]. Nevertheless, the excessive prevalence of kidney stones and high recurrence rates make it questionable whether to perform CT and put children at risk of frequent radiation exposure in every case of possible suggestive symptoms [19]. Children are particularly at risk of developing radiation-associated pathologies because of their longer life expectancy and possible cumulative effects, thus use of non-contrast CT in children should be well-justified. The lack of intra-abdominal fat compared to adults is another significant limitation to the use of CT in young children; this can cause difficulties in evaluating the ureters unless upstream obstruction is present. Furthermore, given the necessity of remaining still during CT, younger children might require sedation [6, 18].

Non-contrast CT, compared to US, has higher sensitivity in detection of small calculi, especially those localised in the ureters [19, 20, 24, 25]. In a study comparing US to non-contrast CT for diagnosing renal stones, Fowler et al. [26] reported an US specificity of 90% but sensitivity of only 24%, though it is important to highlight that the majority of missed calculi were  ≤ 3.0 mm in size. According to multiple studies, such small stones are not clinically significant, do not alter the management and tend to pass spontaneously [20, 24–26]. Thus, even though non-contrast CT is more sensitive in identifying microcalculi, US reveals most clinically relevant stones and given its safety, remains the method of choice in the paediatric population [6, 24]. Use of non-contrast CT in children suspected of having urolithiasis should be limited to certain clinical situations, such as when the child has major colic symptoms with nondiagnostic US and KUB radiography or when other imaging modalities are insufficient for guiding surgical intervention. If necessary, non-contrast CT should always be used with an ultra-low-dose protocol to minimise radiation exposure [10, 18, 19, 24, 25].

### Other modalities

Magnetic resonance (MR) urography is a safe and sensitive (82–100%) method of imaging urolithiasis, visualising stones as signal voids and revealing potential pelvicalyceal dilatation without ionising radiation exposure. MR urography provides detailed anatomical information about the kidneys and collecting system, including three-dimensional (3-D) visualisation, allowing for identification of any urinary tract malformation. Furthermore, MR urography enables evaluation of renal function by assessing parenchymal contrast agent uptake and urine excretion. Despite strong advantages, MR urography accounts for only about 2% of imaging studies in children with suspected kidney stone disease because of its high cost, sedation requirements, long image acquisition times and limited availability. It is sometimes recommended as a complementary method when US is negative [9, 10, 12, 27, 28].

Functional imaging studies such as radionuclide scan with dimercaptosuccinic acid (DMSA) or single photon-emission CT (SPECT-CT) with DMSA can be used in select children, especially those with urolithiasis complicated by UTI and a high possibility of renal scarring. DMSA SPECT-CT can play a role in cases of complex renal calculi, allowing identification of anatomical details of the urinary tract and the number, location and size of stones, as well as providing information about renal parenchymal function. A recent study by Robinson et al. [1] suggested an association between urolithiasis and long-term renal scarring, leading to abnormal DMSA scan results in about 60% of children tested. Thus, the authors recommended considering DMSA scan in children with significant kidney stones or signs of pelvicalyceal systems obstruction.

In recent years, there has been continual progress in the application of artificial intelligence (AI) in diagnostics and management of urolithiasis. Future implementation of AI could lead to improved decision-making and procedural outcome prediction, increased patient safety and more personalised management [29].

## Complications

**Fig. 3:**
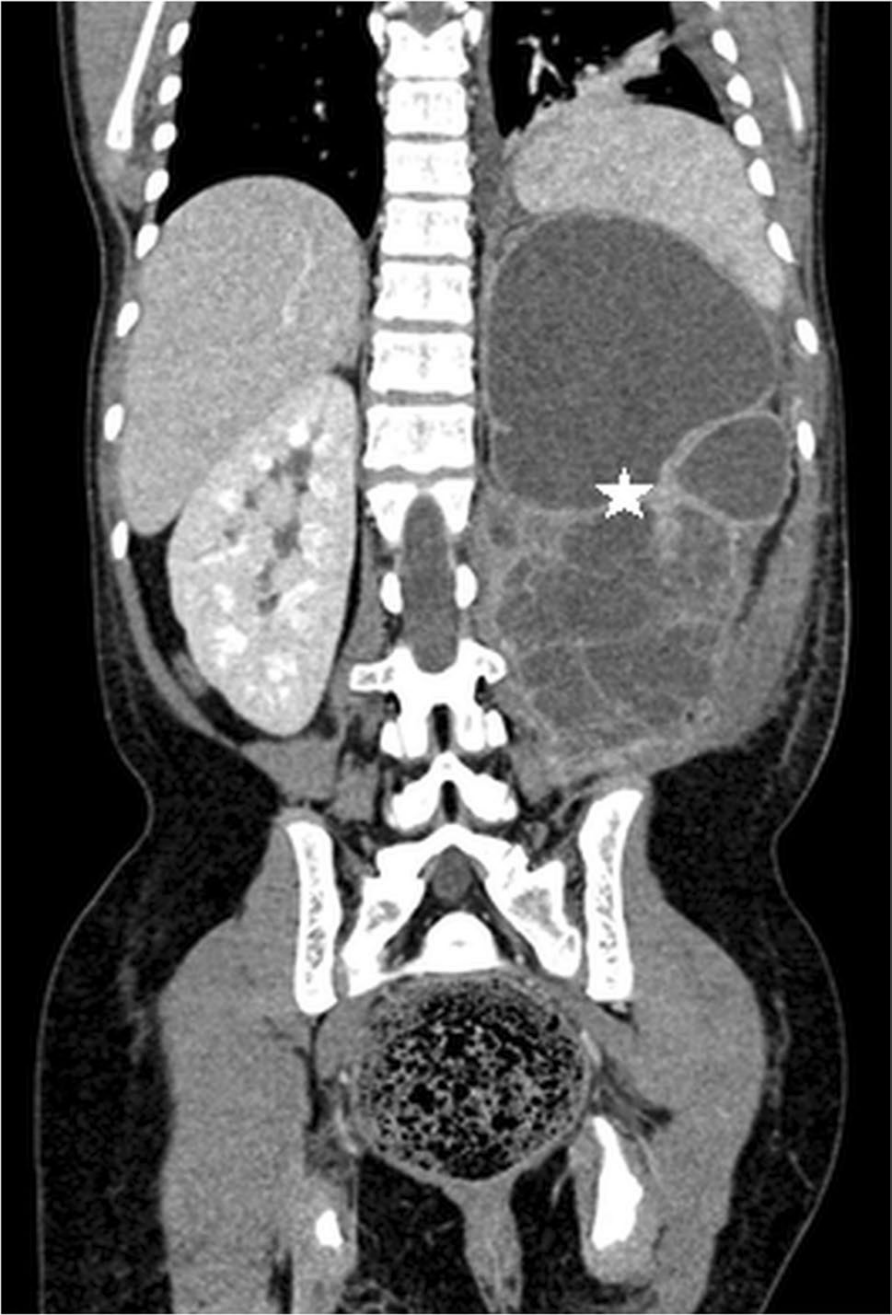
Pyonephrosis in a 13-year-old boy. Coronal contrast-enhanced CT shows a large multicystic lesion with destruction of the left kidney parenchyma (*star*) corresponding to left-side pyonephrosis as a complication of severe obstruction of the pelvicalyceal system

Numerous possible complications arise from urolithiasis, particularly in children with delayed diagnosis and treatment. Khan et al. [30], in their study of children with acute renal colic, reported acute renal failure as the most common complication, observed in as many as 33% of the children. Chronic renal failure may also occur, especially in children with bilateral or recurrent calculi [30, 31]. As a cause of obstruction and pelvicalyceal dilatation, kidney stones are associated with higher risk of urinary tract infections, including recurrent UTIs, chronic pyelonephritis, pyonephrosis (Fig. 3) and sepsis [32–34]. Identifying any signs of infection, such as echogenic debris or gas shadows in the collecting system, thickening of renal pelvic wall, focal areas of abnormal parenchymal echogenicity or areas of reduced vascularity, is crucial. Perinephric fat stranding or formation of parenchymal or perinephric abscess might be seen. Life-threatening forms of UTI such as xanthogranulomatous pyelonephritis with enlargement of the kidney and distortion of its outline or emphysematous pyelonephritis with gas in the collecting system and renal parenchyma combined with parenchymal destruction and areas of necrosis or abscess are uncommon [35, 36]. Single cases of nephrobronchial, psoas muscle or cutaneous fistula formation resulting from stone-associated xanthogranulomatous pyelonephritis have been described [37, 38]. Taşkınlar et al. [39] reported a unique case of spontaneous rupture of the renal pelvis with perirenal urinoma formation caused by a calculus in a previously healthy 18-month-old girl.

## Management

Appropriate management of symptomatic urolithiasis in paediatric patients depends on a few factors, specifically size, location and composition of calculi. The child’s general health, comorbidities and complications, kidney function, anatomical variations of the urinary tract, and the local availability of treatment and experience of physicians must all be considered [40, 41]. Most children with uncomplicated urolithiasis and small stones that are likely to pass spontaneously (< 4–5 mm) do not require urological intervention.

Conservative treatment includes adequate hydration and increased fluid intake, pain control and medical expulsive therapy to relax ureteric smooth muscle and facilitate stone passage [18, 41]. Given the common underlying metabolic abnormalities and very high risk of kidney stone recurrence, children require a thorough metabolic evaluation and follow-up examinations. Change in diet, or long-term pharmacological treatment might be required. Interventional treatment of urolithiasis is generally advised in cases of severe obstruction and surgical decompression (e.g., acute renal failure, infection, obstructed solitary functioning kidney) or unsuccessful pharmacological therapy [41]. Indications and surgical techniques in paediatric patients are similar to those in adults, with the need for general anaesthesia in children being the most important difference. Minimally invasive urological procedures are considered safe and effective; thus, open surgery should be limited to select children with large stones, congenital abnormalities or complications [18, 40].

**Fig. 4:**
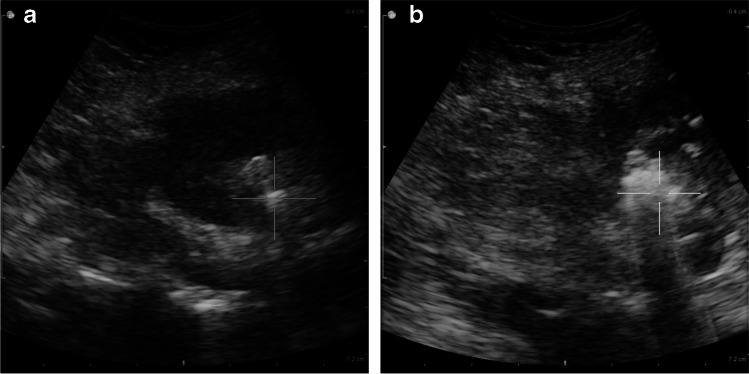
Extracorporeal shock wave lithotripsy in a 15-year-old boy. **a** Transverse US of the right kidney immediately before the procedure shows a large calculus in the pelvicalyceal system. **b** Postoperative transverse image shows fragmentation of the stone into smaller pieces likely to pass spontaneously through the ureter. Case courtesy of Prof. Philippe Petit, Marseille, France

**Fig. 5:**
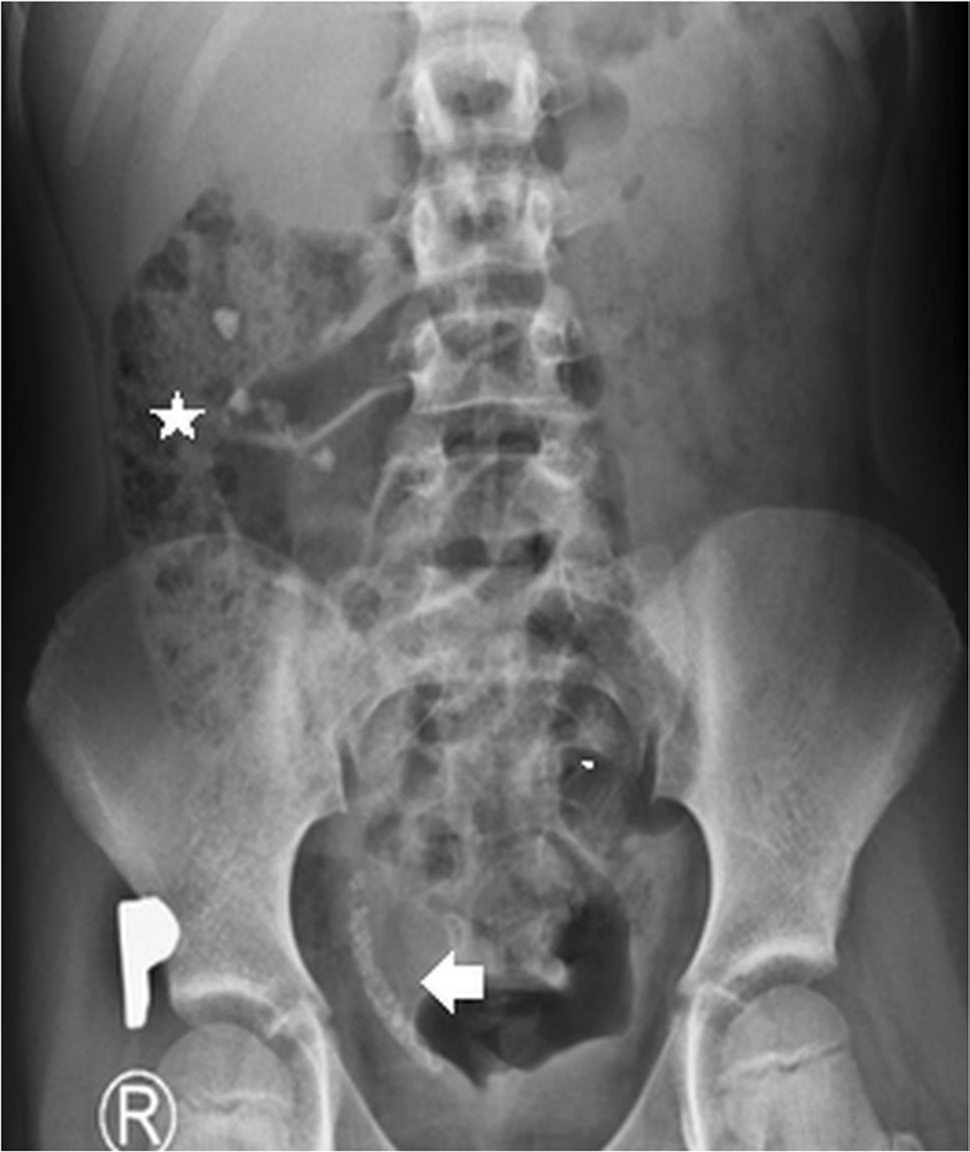
“Steinstrasse” in a 12-year-old boy. Anteroposterior kidney, ureter and bladder radiograph shows steinstrasse, or a column of stone fragments in the distal part of the right ureter (*arrow*) as a complication of extracorporeal shock wave lithotripsy. Note stone fragments in the kidney and right ureter (*asterisk*). Case courtesy of Prof. Philippe Petit, Marseille, France

Extracorporeal shock wave lithotripsy is said to be the least invasive and safest interventional treatment method for proximal stones in children. In this procedure, shock waves are used to fragment the stones into small enough pieces to pass through the ureter (Fig. 4). Additional ureteric stenting is rarely needed [40, 42]. Extracorporeal shock wave lithotripsy is recommended for upper urinary tract stones of diameter  ≤ 1.5–2 cm because its effectiveness is inversely proportional to stone size and decreases with lower calyx or ureteric calculi location. Stone-free rates after the procedure are high, ranging from 57% to 92% [18, 40, 42]. Although extracorporeal shock wave lithotripsy is generally considered safe, it can be complicated by haematuria, infection or “steinstrasse” (German for “stone street”), wherein a column of stone fragments forms and blocks the ureter (Fig. 5), with uncertain long-term consequences in children [40, 41].

Ureteroscopy involves placing a ureteroscope through the bladder to aid the performance of lithotripsy or removal of calculi. It is recommended primarily in children with small ureteric stones, and has high estimated stone-free rates of 93–100% [40, 42]. Up to 7% of patients have ureteroscopy complications such us haematuria, infection, ureteric stricture or ureteric perforation [42]. However, as suggested by Nerli et al. [43], because of the recent development of miniaturised and more durable ureteroscopes, ureteroscopy can be considered safe and the procedure of choice for ureteric and select renal pelvic calculi, even in the youngest children. Follow-up US examination is recommended 2–4 weeks after ureteroscopy [42].

**Fig. 6:**
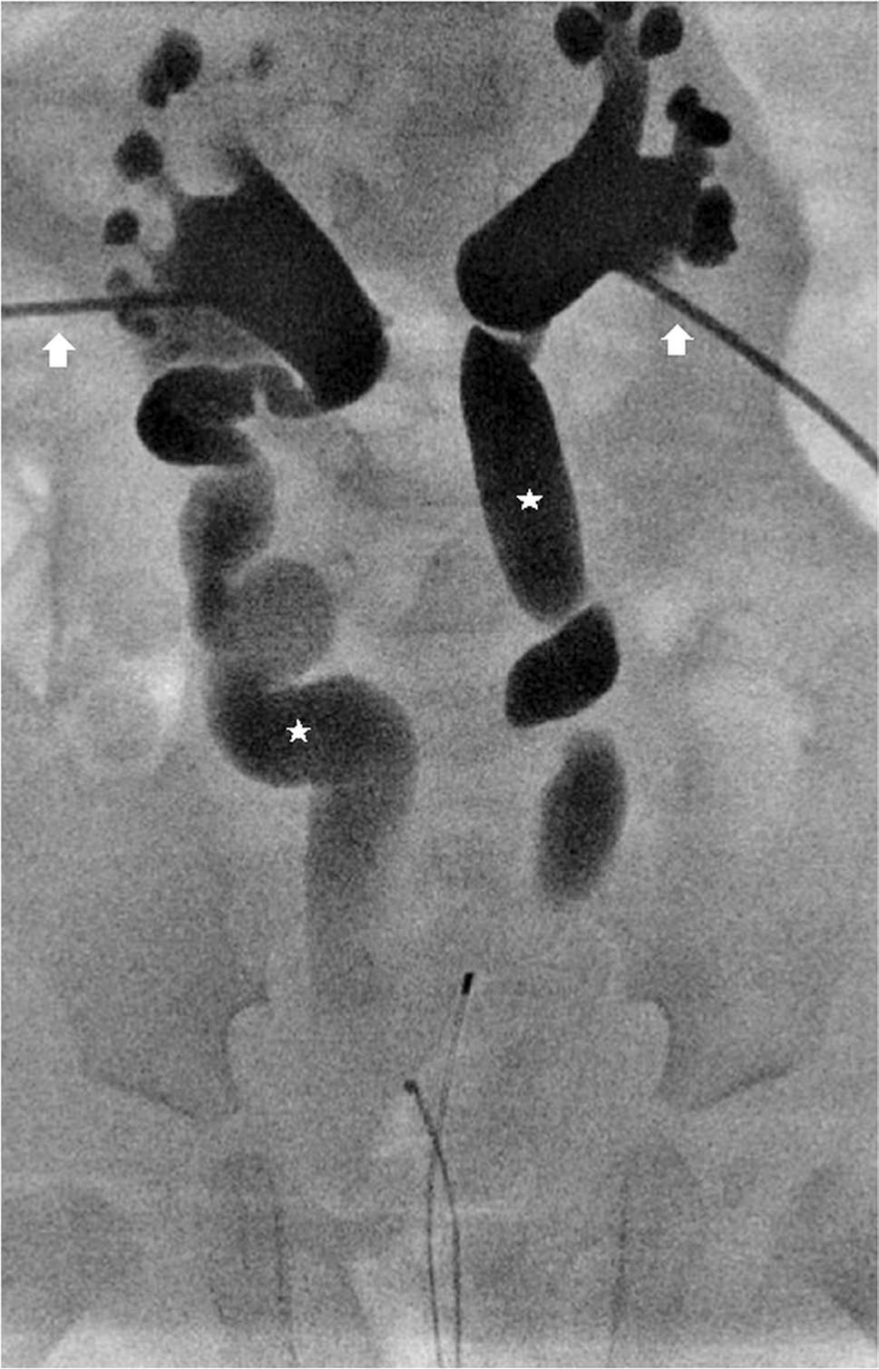
Double nephrostomy performed for bilateral stenosis post ureteral reimplantation in a male neonate. Anteroposterior radiograph with contrast agent administered through nephrostomy tubes (*arrows*) confirms the correct localisation of the catheters in the pelvicalyceal systems and shows dilatation of the urinary tract with enlarged ureters (*stars*). Case courtesy of Pr. Philippe Petit, Marseille, France

**Fig. 7:**
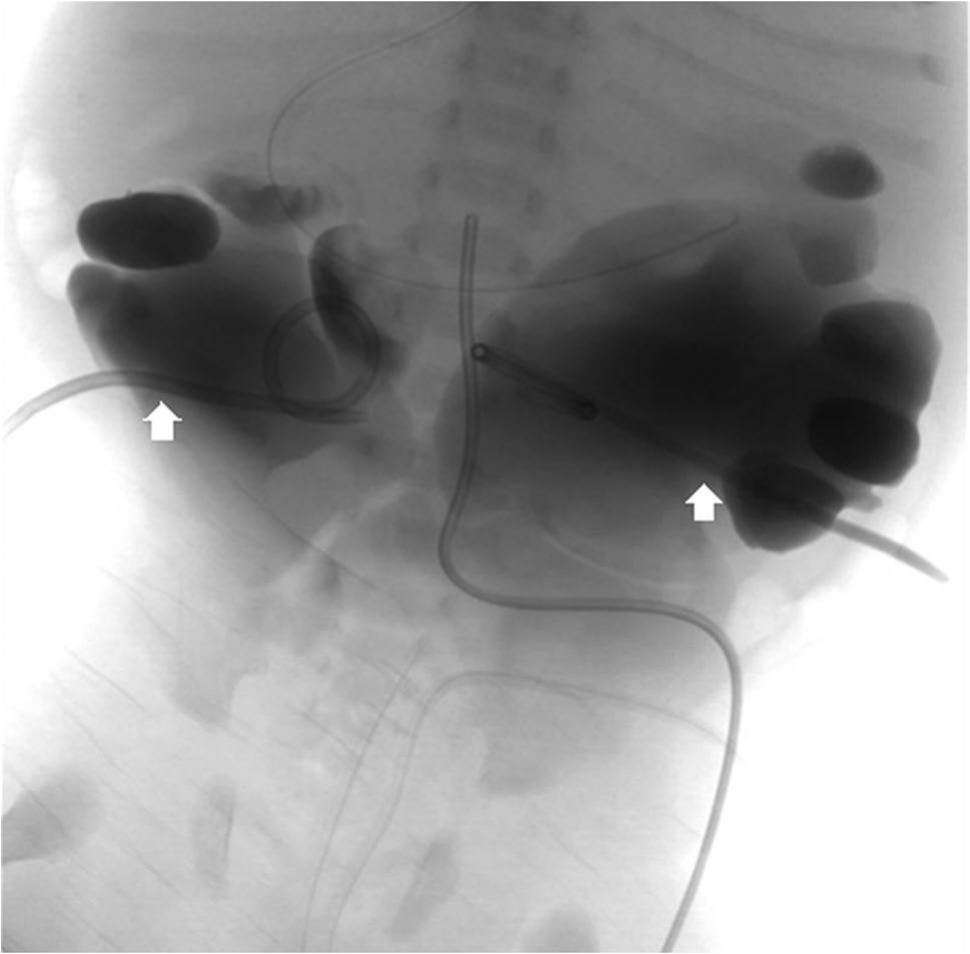
Severe congenital stenotic megaureters causing acute renal failure in a male neonate (different patient from Fig. 6). He underwent a double nephrostomy. Anteroposterior radiograph with contrast agent administered through nephrostomy tubes (*arrows*) shows severe dilatation of the urinary tracts. Case courtesy of Prof. Philippe Petit, Marseille, France

**Fig. 8:**
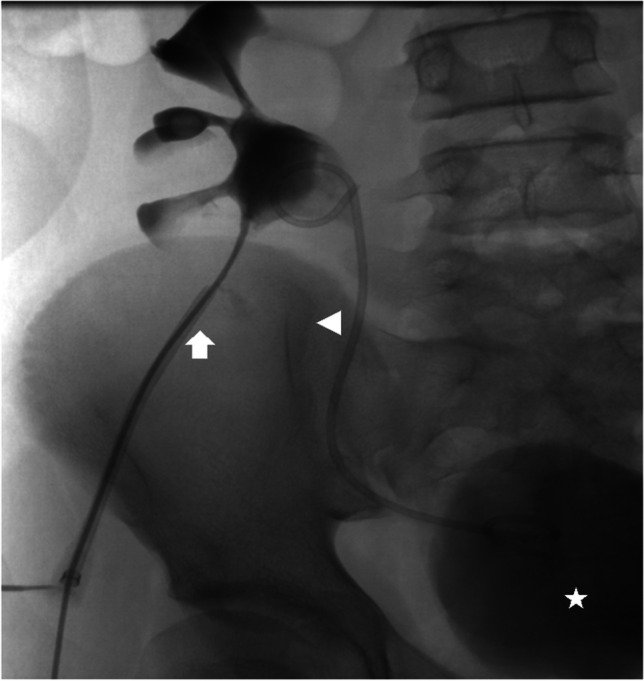
Percutaneous nephrostomy for ureter stenosis of a transplanted kidney in a 17-year-old boy. He underwent stenting of the ureter with a double J catheter to enable urine drainage to the bladder. Anteroposterior radiograph with contrast agent administration through the nephrostomy tube (*arrow*) confirms connection of the pelvicalyceal system to the bladder (*star*) through the double J catheter (*arrowhead*). Case courtesy of Prof. Philippe Petit, Marseille, France

A more invasive procedure, percutaneous nephrostomy, which involves inserting a tract between the skin and renal collecting system, might be indicated in complex cases of urolithiasis to relieve obstruction when transurethral access is impossible (Fig. 6), in cases of acute renal failure affecting a single functioning kidney or both kidneys (Fig. 7), or to provide drainage in children with pyonephrosis or abscess formation. In acutely obstructed and infected kidneys, it is recommended that both percutaneous nephrostomy and transurethral retrograde double J stenting are performed to enable urine drainage into the bladder and to protect the ureters (Fig. 8) [44]. A created passageway from the skin to the renal collecting system can be used to insert urologic devices to crush and remove calculi. The procedure, called percutaneous nephrolithotomy, is reserved for children with large stone burden, significant obstruction, complications or after unsuccessful shock wave lithotripsy. Percutaneous nephrolithotomy is the most effective of the urological interventions for large and complex upper urinary tract stones, with stone-free rate of about 90%; however, severe complications occur in > 10% of patients [18, 41, 42]. Possible complications of the procedure include severe bleeding, collecting system perforation, sepsis or other organ injury [18]. Increasing experience and development of smaller urological equipment have resulted in implementation of mini-, ultra-mini- and micro-percutaneous nephrolithotomy techniques to reduce the risk of complications [45].

## Conclusion

Ultrasound allows for identification of most clinically relevant stones, making it the modality of choice in paediatric urolithiasis. In children with symptoms of significant colic and nondiagnostic US, complementary imaging modalities such as KUB radiography or non-contrast CT might be considered. Management of kidney stones includes dietary, pharmacological and urological interventions depending on stone size, location or type and the child’s condition. Given the very high incidence of underlying metabolic abnormalities and significant recurrence rates in paediatric urolithiasis, a thorough metabolic evaluation and follow-up examinations are of utmost importance.
